# NGS-Integrator: An efficient tool for combining multiple NGS data tracks using minimum Bayes’ factors

**DOI:** 10.1186/s12864-020-07220-7

**Published:** 2020-11-19

**Authors:** Bronte Wen, Hyun Jun Jung, Lihe Chen, Fahad Saeed, Mark A. Knepper

**Affiliations:** 1 Epithelial Systems Biology Laboratory, Systems Biology Center, National Heart, Lung, and Blood Institute, National Institutes of Health, Bethesda, MD USA; 2 Division of Nephrology, Department of Medicine, Johns Hopkins University School of Medicine, Baltimore, MD USA; 3 School of Computing and Information Sciences, Florida International University, Miami, Florida USA

**Keywords:** Efficient data integration, Genome-wide NGS, NGS data analysis, Minimum Bayes factor

## Abstract

**Background:**

Next-generation sequencing (NGS) is widely used for genome-wide identification and quantification of DNA elements involved in the regulation of gene transcription. Studies that generate multiple high-throughput NGS datasets require data integration methods for two general tasks: 1) generation of genome-wide data tracks representing an aggregate of multiple replicates of the same experiment; and 2) combination of tracks from different experimental types that provide complementary information regarding the location of genomic features such as enhancers.

**Results:**

*NGS-Integrator* is a Java-based command line application, facilitating efficient integration of multiple genome-wide NGS datasets. *NGS-Integrator* first transforms all input data tracks using the complement of the minimum Bayes’ factor so that all values are expressed in the range [0,1] representing the probability of a true signal given the background noise. Then, *NGS-Integrator* calculates the joint probability for every genomic position to create an integrated track. We provide examples using real NGS data generated in our laboratory and from the mouse ENCODE database.

**Conclusions:**

Our results show that *NGS-Integrator* is both time- and memory-efficient. Our examples show that *NGS-Integrator* can integrate information to facilitate downstream analyses that identify functional regulatory domains along the genome.

**Supplementary Information:**

The online version contains supplementary material available at 10.1186/s12864-020-07220-7.

## Background

Genome-wide next-generation sequencing (NGS) can provide information about binding of proteins to DNA, chromatin modifications, and chromatin accessibility. These factors are under intense study because they determine what genes are expressed in a given cell type and how gene expression is regulated in different physiological or pathophysiological states. In general, as with any experimental data, genome-wide NGS data has background noise, random and systematic errors that can obscure the features being investigated. Therefore, multiple replicates are needed to maximize confidence and consensus in feature identification. It is essential to replicate multiple NGS experiments for confidence in the produced data. However, approaches to utilization of multiple data tracks for feature identification has been limited largely to visualization of superimposed [1] or stacked data tracks. Peak-calling tools [2–5] are typically applied to individual replicates and not the aggregate data from multiple tracks.

In this paper, we propose an efficient method for integrating multiple tracks for data averaging of multiple replicates. Our proposed method, called *NGS-Integrator*, transforms genome-wide NGS data for identification of genomic regulatory elements and chromatin modifications (e.g. ChIP-Seq, ATAC-Seq, and Bisulfite-Seq) to a probability vector using the complement of the minimum Bayes’ factor for each track, followed by calculation of joint probabilities for the tracks as a function of position along the genome. The integrated tracks can then be used as input to peak calling programs to identify specific features with greater specificity.

## Methods

*NGS-Integrator* combines NGS data tracks from experiments that identify genomic regulatory elements and chromatin modifications (e.g. ChIP-Seq, ATAC-Seq, and Bisulfite-Seq), but not RNA-Seq data. A genome-wide NGS track is a vector R = [r_*i*_, *i*] where r_*i*_ represents a measured value at nucleotide base position *i* along the length of DNA representing the genome. Usually, but not always, r_*i*_ represents a discrete value, e.g. the number of sequencing reads that overlap the position. In general, values of r_*i*_ can range from 0 to infinity. Conceptionally, the value r_*i*_ gives information about the likelihood of a given feature being present, e.g. binding of a particular transcription factor (TF) from ChIP-Seq data, when considered in the context of the intrinsic background noise, n_*i*_, in the signal. To transform the data in terms of probability of a feature, we can use the complement of the minimum Bayes factor,

$$ {\mathrm{p}}_i=1-\exp \left[-\frac{{\mathrm{z}}_i^2}{2}\right], $$

where z_*i*_ equals r_*i*_/n_*i*_. With two data tracks *k* = 1,2, a probability vector that combines information from both can be obtained by calculating the joint probability at each *i* (P_*i*_) as

$$ {\mathrm{P}}_i={\Pi}_{k=1}^2{p}_k $$

### Implementation

#### Algorithm

**Fig. 1:**
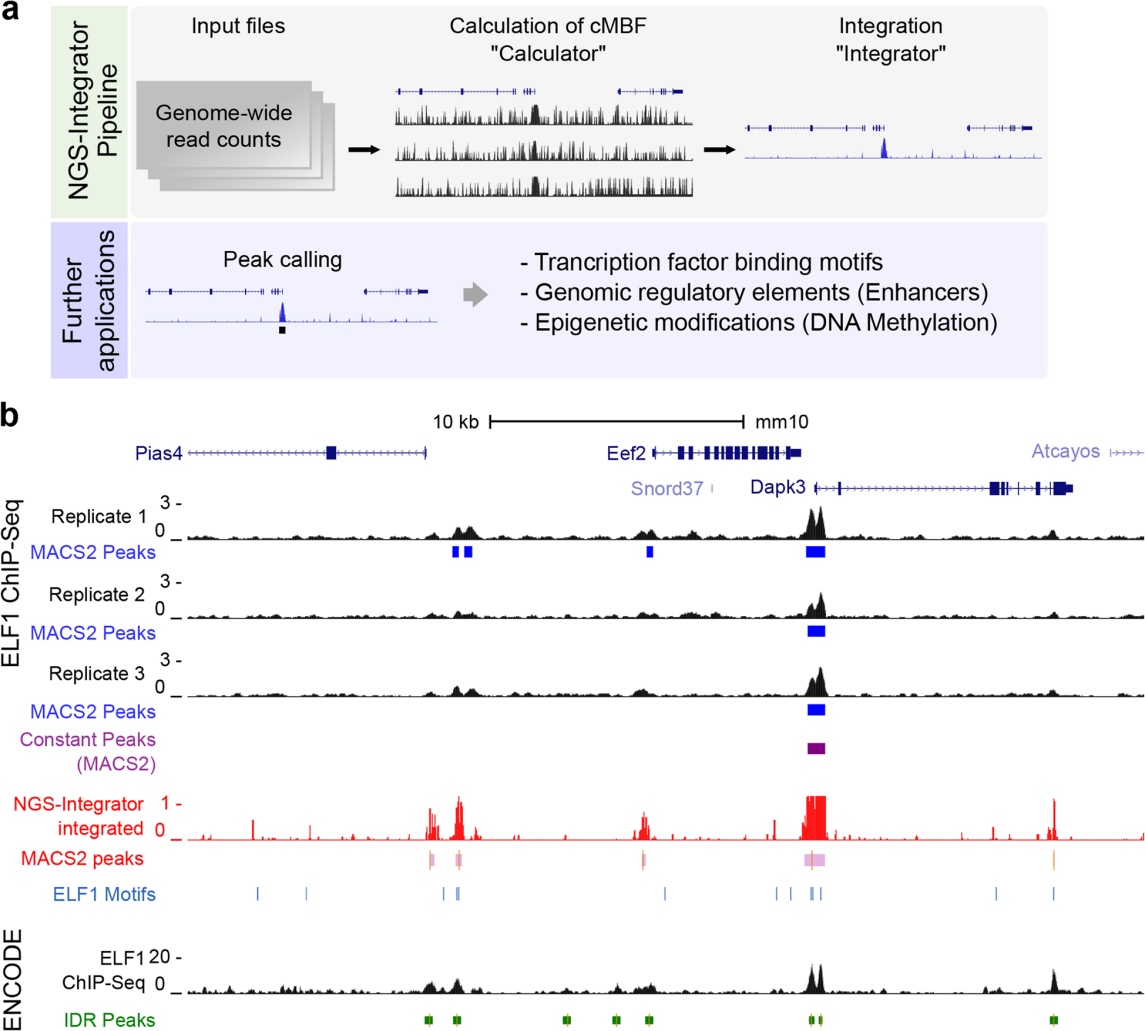
Workflow of NGS-Integrator and example of application in analysis of ChIP-Seq data. **a** *NGS-Integrator* pipeline and further applications for integration of multiple genome-wide DNA sequencing data. **b** Identification of genomic binding sites for transcription factor ELF1 near *Eef2* gene. Three replicates of ChIP-Seq data for ELF1 (E74-like factor 1), which is an Ets family TF, were generated in mpkCCD cells. The replicate data tracks were integrated (window size for background noise calculation: 10 kb with 2× median across the window). The *NGS-Integrator* integrated track (red track) was generated from the three replicates and consensus ELF1 binding motifs (*Homer* motif database) were indicated by blue lines. ELF1 ChIP-Seq data (ENCSR033OWC) from the mouse ENCODE database with fold change of signal over control track and its optimal irreproducible discovery rate (IDR) threshold peaks (green bars, orange bars indicate summits of peaks) are displayed for comparison. Peaks identified from a conventional peak calling tool *MACS2* were labeled by blue bars for peaks from each replicate and purple bar for the conserved peaks across the replicates. In addition, peaks identified using *MACS2* (bdgcallpeak, cutoff > 0.5) was also shown below the *NGS-Integrator* integrated track (red). All data tracks were displayed on the *UCSC* Genome Browser with mouse genome mm10

Computationally, *NGS-Integrator* consists of two elements (Fig. 1a). The *Calculator* element calculates the complement of the minimum Bayes factor (cMBF) at each position *i*, estimating n_*i*_ as the median of the r_*i*_ values across a window of *i* values straddling the position at which p_i_ is being calculated. This calculation assumes that the features being detected are relatively sparsely represented along the genome, allowing the median to be representative of the true background noise level. The *Integrator* element calculates the joint probability between the two NGS tracks P_*i*_ based on the respective p_*i*_ vectors. When integrating multiple tracks, sequential dual-input calculations are performed, based on the commutativity of the joint probability operation. Also, it is obvious that the computational time complexity of the algorithms is O(kw) where k is the number of nucleotides in the genome and w is the size of the window. The memory-complexity of the algorithm is a linear function of k.

#### Input and output

For calculation of genome-wide minimum Bayes’ factors from the read depth, *NGS-Integrator* requires a bed file containing coverage (number of the sequence reads at each nucleotide) generated from standard tools, such as *samtools* (depth) and *bedtools* (coverage, genomecov). The input file should contain 4 fields, chromosome number, start region, end region and coverage value. The output of *NGS-Integrator* as a BED file contains cMBF values at each nucleotide position across the whole genome. The output BED file can be easily modified to bedGraph or BigWig file format to be visualized on genome browsers. The output of *NGS-Integrator* could also be used to identify overlapping regions across multiple genome-wide NGS data types (Fig. 1a).

#### Datasets for integration analyses using *NGS-Integrator*

Three replicates of ChIP-Seq for the transcription factor ELF1 (unpublished) using an ELF1 antibody (sc-631, Santa Cruz Biotechnology) done in mouse kidney cortical collecting duct cell mpkCCD cells as described previously [6] were used to generate input BED files with genome-wide coverage (10 bp-scale bin). Coverage across the whole genome was estimated by *bedtools* (coverage) from BAM files. To compare with our ELF1 ChIP-Seq data, public data for ELF1 ChIP-Seq data in erythroleukemia cells (ENCSR033OW) with fold change of signal over control track and its optimal irreproducible discover rate threshold peaks was downloaded from the mouse ENCODE database (https://www.encodeproject.org/).

Four replicates of ATAC-Seq data (GSE108786) were generated in mpkCCD cells as described previously [6]. Datasets of ChIP-Seq data for histone H3K27Ac (GSE95009) and RNA Polymerase II (GSE79584) were used from previous published data [7, 8]. Genome-wide coverage of every 10 bp-scale bin as an input bed file of each replicate was generated by *bedtools* (coverage) from BAM files.

Multiple ChIP-Seq datasets (GSE29218) published as part of the mouse ENCODE project were used to examine integration of multiple types of ChIP-Seq data. The ChIP-Seq datasets for P300 (GSM723018), RNA Polymerase II (GSM723019) and H3K27Ac (GSM851278) were generated in Bruce4 mouse embryonic stem cells (mESCs) by Dr. Bing Ren’s laboratory [9]. Two replicates from each ChIP-Seq dataset were used for integration using *NGS-Integrator*.

## Results

### Example 1:Integration of multiple-replicate transcription factor ChIP-Seq data

To provide an example of an application of *NGS-Integrator* for genome-wide NGS data for multiple replicates of the same experiment, we processed previously unpublished datasets obtained from antibody-based chromatin immunoprecipitation followed by NGS (ChIP-Seq) using an antibody to the transcription factor ELF1 (Fig. 1b). Based on technical limitations, especially in the antibody-based immunoprecipitation step, ChIP-Seq data for TF binding site identification often includes significant background noise that obscures specific binding sites. In this example, we used three replicates of ELF1 ChIP-Seq data generated in mouse kidney cortical collecting duct (mpkCCD) cells [6, 10]. Figure 1 shows a genomic region on the *UCSC* genome browser that includes the *Eef2* gene. The replicates are shown along with peaks identified with *MACS2* [11] for each. Only one peak is common to all three replicates. Integration of three replicate datasets using *NGS-Integrator* is shown as the red track. When this track was used as input to *MACS2*, additional peaks were called, many of which contain typical ELF1 binding motifs (ANCCGGAAGT) identified with *Homer* 4.9 (blue lines, Fig. 1). For comparison, we showed ELF1 ChIP-Seq data reported in ENCODE (ENCSR033OWC) for murine erythroleukemia (MEL) cells along with called peaks (*IDR* peaks) indicated by green bars. ELF1 binding from the present study is consistent with ELF1 binding in the ENCODE data even though the cell types are different. We provide an additional example of the use of *NGS-Integrator* to integrate ChIP-Seq data in Supplementary Fig. 1, mapping genomic binding sites of the TF SNAIL1 in human LS174T colorectal cancer cells [12].

### Example 2:Integration of multiple replicates of ATAC-Seq data

**Fig. 2:**
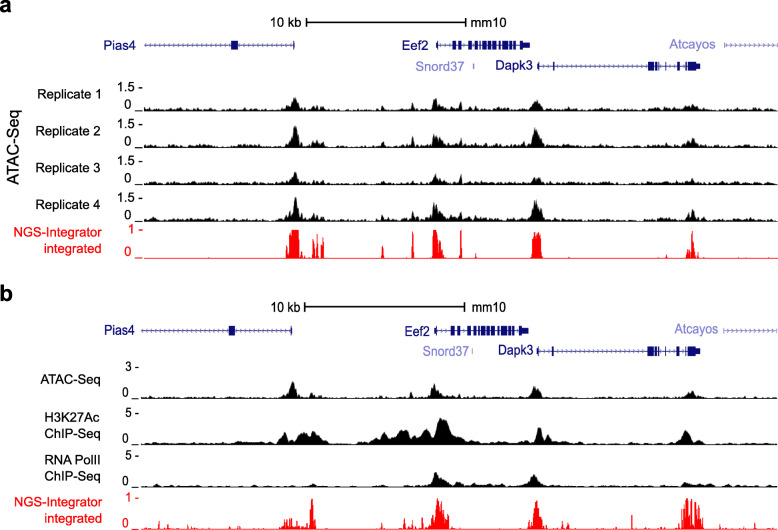
Examples of data integration for homogeneous and heterogeneous NGS data. **a** Identification of open chromatin regions near *Eef2* gene using ATAC-Seq. Tracks for replicates of ATAC-Seq data from mpkCCD cells were integrated into a single track using *NGS-Integrator* (window size: 10 kb, background: 2× median across the window). *Eef2* gene, coding for Elongation Factor 2 (EF-2), is a housekeeping gene and mainly involved in the GTP-dependent ribosomal translocation step during translation elongation. Original data tracks in black; integrated data track in red. **b** Identification of active enhancer regions near *Eef2* gene using multiple NGS techniques. Three different NGS datasets were generated using data from ATAC-Seq, ChIP-Seq for histone H3K27Ac and ChIP-Seq for RNA Polymerase II in mouse kidney cortical collecting duct cell mpkCCD. The datasets were integrated to identify potential regulatory elements (window size: 10 kb, background: 2× median across the window). Original data tracks in black; integrated track in blue. All data tracks were displayed on the *UCSC* Genome Browser with mouse genome mm10

ATAC-Seq (Assay for Transposase-Accessible Chromatin using Sequencing) is a recently developed method to map open chromatin regions across the genome [13] and is able to identify regulatory DNA elements where transcriptional regulators bind. We previously applied ATAC-Seq in mpkCCD cells to identify active regulatory DNA elements near the *Aqp2* gene, which codes for the water channel protein aquaporin-2 [6]. Here, four replicates of ATAC-Seq data from mpkCCD cells were integrated (Fig. 2a) as another example of homogeneous data integration. Data integration using *NGS-Integrator* allows the data to be summarized in a single track with improved overall signal-to-noise discrimination (red track, Fig. 2a).

### Example 3:Combining data from RNA polymerase II ChIP-Seq, histone H3K27Ac ChIP-Seq, and ATAC-Seq to predict locations of enhancers

As a third example, heterogeneous data integration was done using three types of data expected to overlap at active enhancer sites, viz. ATAC-Seq, ChIP-Seq for histone H3 acetylation at Lysine 27 (H3K27Ac) and ChIP-Seq for RNA polymerase II (RNA Pol II), all from mouse mpkCCD cells (Fig. 2b). Although these three features are known to overlap at enhancers [14], none alone are adequate to precisely identify the boundaries of active enhancers. Typically, sequences within identified enhancers are analyzed to identify putative TF binding sites. By sharpening enhancer boundaries, *NGS-Integrator* can narrow the list of putative TFs that need to be studied further to understand the regulation of a particular gene.

### Example 4:Combining data from P300, RNA polymerase II, histone H3K27Ac ChIP-Seq datasets to predict locations of active regulatory elements

**Fig. 3:**
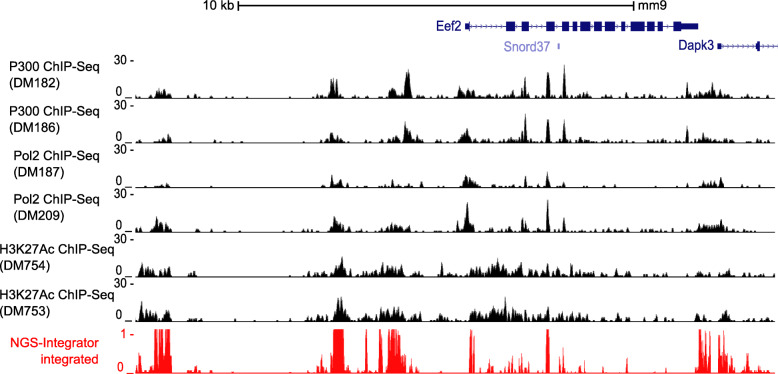
Data integration for multiple ChIP-Seq data from the public mouse ENCODE database. Identification of active regulatory regions near *Eef2* gene using multiple ENCODE ChIP-Seq data (GSE29218) generated in mouse Embryonic Stem Cell (mESC) line Bruce4. Tracks from three datasets with two replicates of each ChIP-Seq were integrated into a single track using *NGS-Integrator* (window size: 10 kb, background: 2× median across the window). The genome browser for *Eef2* gene on mouse reference genome mm9 was shown. Original data tracks in black; integrated data track in red

To test integration of a large number of datasets, *NGS-Integrator* was applied to ChIP-Seq datasets published by Dr. Bing Ren’s laboratory. Total 6 ChIP-Seq datasets for the histone acetyltransferase P300, RNA Pol II and Histone H3K27Ac (two replicates per target protein) generated from mouse embryonic stem cell line, Bruce4, were used to calculate cMBFs from each dataset and combine all 6 datasets into one track with *NGS-Integrator* (Fig. 3), revealing active regions surrounding *Eef2* including one at the transcriptional start site.

### Performance characteristics

**Fig. 4:**
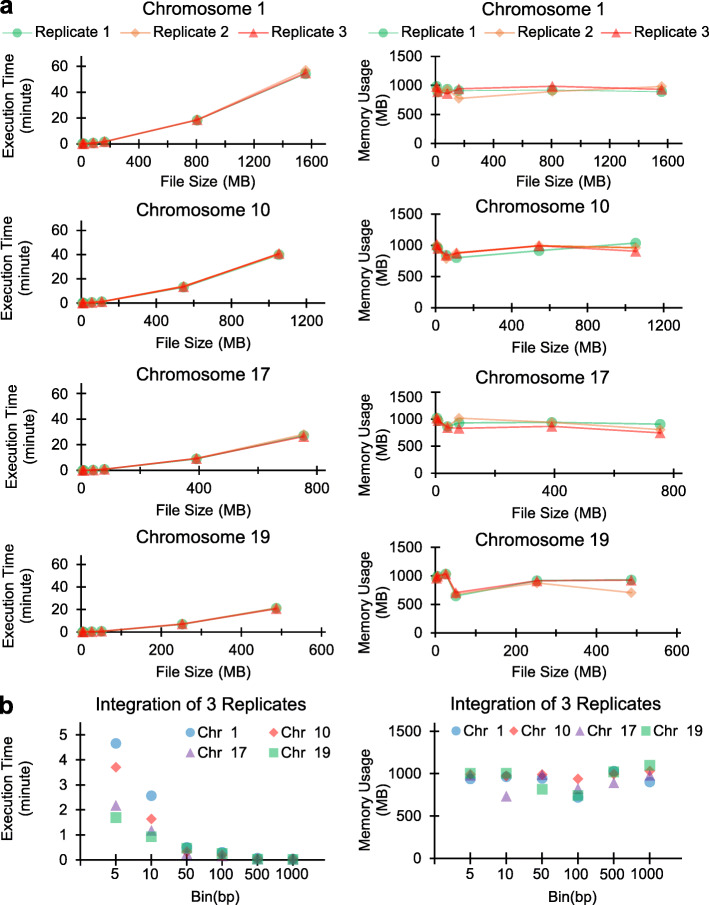
Estimation of execution time and memory usage of *NGS-Integrator* ‘Calculator’ and ‘Integrator’. **a** Execution time (left panel) and memory usage (right panel) of *NGS-Integrator* ‘Calculator’ was assessed from different sizes of chromosomes (chromosome 1, 10, 17, and 19) with multiple scale bins (1000 bp, 500 bp, 100 bp, 50 bp, 10 bp, and 5 bp). ‘Calculator’ was tested as default setting (window size: 10000 bp; number to replace zero: 0.1). **b** Execution time and memory usage of *NGS-Integrator* ‘Integrator’ estimated from cMBFs of three replicates in each chromosome size. The performance analysis was accomplished using 2 Hexa core Intel Xeon CPU E5–2620 running at 2.40GHz with 32 KB of L1 instruction cache, 32 kb of L1 data cache, 256 kb of L2, and 15,360 kb of L3 cache. The Linux system was equipped with 48GB of RAM memory and operating system was 3.13.0–48 generic Ubuntu

Figure 4 shows execution time and memory usage for a range of input file sizes for the *Calculator* module (Fig. 4a) and the *Integrator* module of *NGS-Integrator* (Fig. 4b). To vary input file sizes, multiple scale bins (1000 bp, 500 bp, 100 bp, 50 bp, 10 bp and 5 bp) were generated in four different mouse chromosomes (1, 10, 17, and 19) through the coverage calculation using *bedtools* prior to application of *NGS-Integrator*. As shown in Fig. 4a, the execution time increases with a linear-trend with increasing size of the input file. The memory consumption for given set of files was also noted to be consistent with increasing size of the data. Figure 4b shows that decreasing the file size for integrating output files from the *Calculation* module corresponds to a sharp decrease in the execution time, as expected. Complexity analysis of the algorithms suggests that it can operate in linear time and space with respect to the length of the genome, consistent with the results shown here.

## Conclusions

In this paper we have proposed, designed and implemented easy-to-use software called *NGS-Integrator* which allows integration of multiple genome-wide NGS data tracks. Our software is implemented in Java and allows integration of data obtained either from multiple datasets of the same technique or data sets derived from different, but complementary, techniques. As shown in the examples, *NGS-Integrator* can integrate information to facilitate downstream analyses that identify functional domains along the genome. In the *Calculator* module, the NGS data are transformed to range [0,1] through use of the complement of the minimum Bayes’ factor. Fundamentally, any data transformation that yields values in the range [0,1] and has a positive monotonic relation to the input data would work. The main reason for the choice of the minimum Bayes’ factor is the direct use of the background noise for the calculation, which is easily estimated for sparse data as described. We expect that *NGS-Integrator* will be very useful for a wide variety of experimental studies where multiple replicates of genome-wide data need to be integrated into a single track.

## Availability and requirements

Project name: NGS-Integrator.

Project home page: https://hpcwebapps.cit.nih.gov/ESBL/NGS-Integrator/.

Archived version: GitHub (https://github.com/ESBL/NGS-Integrator).

Operating system(s): Platform independent.

Programming language: Java.

Other requirements: Java 1.7 or higher.

License: Open source.

## Supplementary Information

**Additional file 1: Fig. S1.** Examples of data integration using *NGS-Integrator* to identify genomic binding sites for transcription factor SNAIL1-HA in human LS174T colorectal cancer cells. a and b Two replicates of ChIP-Seq data for SNAIL1-HA obtained from GSE127183 were integrated (window size for background noise calculation: 10 kb with 2× median across the window). The *NGS-Integrator* integrated track (red track) was generated from the two replicates and peaks identified using *MACS2* (bdgcallpeak, cutoff > 0.5) was also shown below the *NGS-Integrator* integrated track (red). All data tracks were displayed on the *UCSC* Genome Browser with human genome hg19.

## Data Availability

The datasets analyzed during the current study are available in the GEO: ATAC-Seq data (GSE108786), ChIP-Seq data for histone H3K27Ac (GSE95009), and RNA Polymerase II (GSE79584). The datasets of ELF1 ChIP-seq analyzed during the current study are available from the corresponding author on request.

## Abbreviations

NGS: Next-generation sequencing
TF: Transcription factor
cMBF: Complement of the minimum Bayes factor
ChIP-seq: Chromatin immunoprecipitation followed by DNA sequencing
ATAC-seq: Assay for Transposase-Accessible Chromatin using sequencing
H3K27Ac: Histone H3 acetylation at lysine 27
RNA Pol II: RNA polymerase II

## References

[1] An J, Lai J, Wood DL, Sajjanhar A, Wang C, Tevz G (2015). RNASeqBrowser: a genome browser for simultaneous visualization of raw strand specific RNAseq reads and UCSC genome browser custom tracks. BMC Genomics.

[2] Feng J, Liu T, Qin B, Zhang Y, Liu XS (2012). Identifying ChIP-seq enrichment using MACS. Nat Protoc.

[3] Rozowsky J, Euskirchen G, Auerbach RK, Zhang ZD, Gibson T, Bjornson R (2009). PeakSeq enables systematic scoring of ChIP-seq experiments relative to controls. Nat Biotechnol.

[4] Xu J, Zhang Y (2012). A generalized linear model for peak calling in ChIP-Seq data. J Comput Biol.

[5] Ji H, Jiang H, Ma W, Johnson DS, Myers RM, Wong WH (2008). An integrated software system for analyzing ChIP-chip and ChIP-seq data. Nat Biotechnol.

[6] Jung HJ, Raghuram V, Lee JW, Knepper MA (2018). Genome-wide mapping of DNA accessibility and binding sites for CREB and C/EBPbeta in vasopressin-sensitive collecting duct cells. J Am Soc Nephrol.

[7] Sandoval PC, Claxton JS, Lee JW, Saeed F, Hoffert JD, Knepper MA (2016). Systems-level analysis reveals selective regulation of Aqp2 gene expression by vasopressin. Sci Rep.

[8] Isobe K, Jung HJ, Yang CR, Claxton J, Sandoval P, Burg MB (2017). Systems-level identification of PKA-dependent signaling in epithelial cells. Proc Natl Acad Sci U S A.

[9] Shen Y, Yue F, McCleary DF, Ye Z, Edsall L, Kuan S (2012). A map of the cis-regulatory sequences in the mouse genome. Nature..

[10] Yu MJ, Miller RL, Uawithya P, Rinschen MM, Khositseth S, Braucht DW (2009). Systems-level analysis of cell-specific AQP2 gene expression in renal collecting duct. Proc Natl Acad Sci U S A.

[11] Zhang Y, Liu T, Meyer CA, Eeckhoute J, Johnson DS, Bernstein BE (2008). Model-based analysis of ChIP-Seq (MACS). Genome Biol.

[12] Beyes S, Andrieux G, Schrempp M, Aicher D, Wenzel J, Anton-Garcia P (2019). Genome-wide mapping of DNA-binding sites identifies stemness-related genes as directly repressed targets of SNAIL1 in colorectal cancer cells. Oncogene..

[13] Buenrostro JD, Giresi PG, Zaba LC, Chang HY, Greenleaf WJ (2013). Transposition of native chromatin for fast and sensitive epigenomic profiling of open chromatin, DNA-binding proteins and nucleosome position. Nat Methods.

[14] Spicuglia S, Vanhille L (2012). Chromatin signatures of active enhancers. Nucleus..

