# Supplementary figures and images for: NGS-Integrator: An efficient tool for combining multiple NGS data tracks using minimum Bayes’ factors
Source: BMC Genomics. 2020 Nov 19;21:806. doi: 10.1186/s12864-020-07220-7 (PMC7678096; doi:10.1186/s12864-020-07220-7)

Supplementary Figure 1

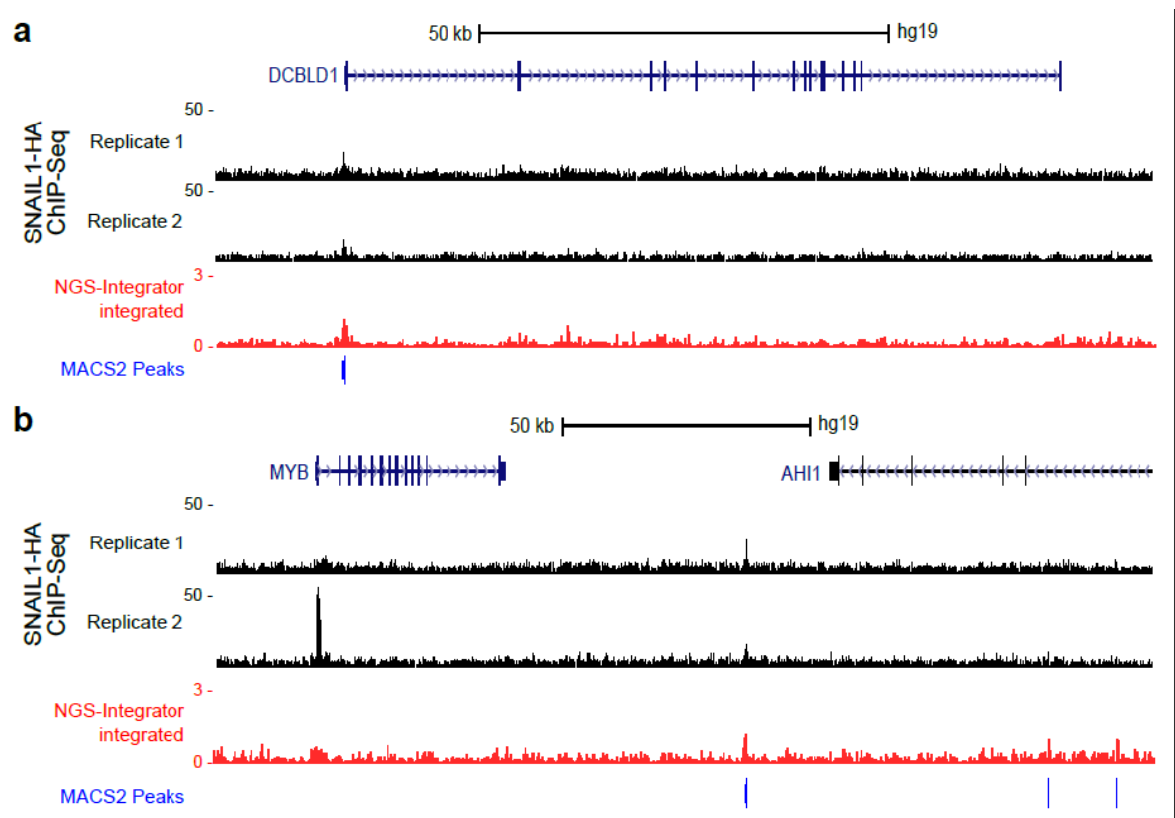

Supplement: Supplementary file 1 — Additional file 1: Fig. S1. Examples of data integration using NGS-Integrator to identify genomic binding sites for transcription factor SNAIL1-HA in human LS174T colorectal cancer cells. a and b Two replicates of ChIP-Seq data for SNAIL1-HA obtained from GSE127183 were integrated (window size for background noise calculation: 10 kb with 2× median across the window). The NGS-Integrator integrated track (red track) was generated from the two replicates and peaks identified using MACS2 (bdgcallpeak, cutoff > 0.5) was also shown below the NGS-Integrator integrated track (red). All data tracks were displayed on the UCSC Genome Browser with human genome hg19. [file 12864_2020_7220_MOESM1_ESM.pdf]
